# The co-existence of NAFLD and CHB is associated with suboptimal viral and biochemical response to CHB antiviral therapy: a systematic review and meta-analysis

**DOI:** 10.3389/fgstr.2024.1333988

**Published:** 2024-01-24

**Authors:** Georgia Zeng, Benjamin R. Holmes, Saleh A. Alqahtani, Upkar S. Gill, Patrick T. F. Kennedy

**Affiliations:** ^1^ Faculty of Medicine, St Vincent’s Clinical School, University of New South Wales, Sydney, NSW, Australia; ^2^ Datasight Ltd, Norwich, United Kingdom; ^3^ Organ Transplant Center of Excellence, King Faisal Specialist Hospital & Research Center, Riyadh, Saudi Arabia; ^4^ Division of Gastroenterology & Hepatology, Johns Hopkins University, Baltimore, MD, United States; ^5^ Barts Liver Centre, Blizard Institute, Barts and The London School of Medicine and Dentistry, Queen Mary University of London, London, United Kingdom

**Keywords:** NAFLD, CHB (chronic hepatitis B), antiviral treatment, treatment efficacy, HBV - hepatitis B virus

## Abstract

**Background and aims:**

Chronic hepatitis B (CHB) and non-alcoholic fatty liver disease (NAFLD) are leading causes of liver-related morbidity and mortality. The interaction between these two disease processes is poorly defined and the impact of NAFLD on HBV-related cirrhosis and HCC remains unclear. The aim of this study was to evaluate the impact of NAFLD on response to antiviral CHB therapy to inform the debate on changing CHB treatment thresholds for these comorbid patients.

**Methods:**

Studies with a minimum of 50 adult CHB patients on nucleoside analogue therapy with or without concurrent NAFLD were identified from PubMed/Medline and EMBASE to February 21, 2023. Data extraction from each study included HBeAg and treatment status, diagnostic method of NAFLD, frequency of monitoring intervals, patient age, gender, grade of hepatic steatosis, BMI and metabolic comorbidities. The outcomes of interest, complete virological response (CVR), biochemical response (BR) and HBeAg loss/seroconversion, were recorded at each available monitoring interval. Comparing CHB-NAFLD and CHB-only groups, pooled odds ratios (OR) and 95% confidence intervals (CI) were calculated using random- or fixed-effects models depending on heterogeneity.

**Results:**

From a search of 470 citations, we identified 32 potentially relevant papers. Overall, 11 studies, comprising 2580 unique patients, met the inclusion criteria of the meta-analysis. CHB-NAFLD patients exhibited significantly lower rates of CVR compared to CHB-only patients. This was demonstrated by an OR of 0.59 (0.38-0.93, p=0.001, I^2 =^ 72%) at 12 months, which tapered off to an OR of 0.67 (0.48-0.95, p=0.02) at 60 months. CHB-NAFLD patients also exhibited significantly lower rates of BR compared to CHB-only patients, as demonstrated by ORs of 0.39 (0.24-0.62, p<0.0001, I^2 =^ 53%) at 12 months and 0.33 (0.17-0.63, p=0.0008) at 24 months.

**Conclusion:**

Patients with concurrent CHB and NAFLD experience delayed CVR to antiviral therapy and more persistent biochemical abnormalities in comparison to patients with CHB only. This supports the argument for earlier antiviral therapy in order to avert CHB complications in these multi-morbid patients, as the global disease burden of NAFLD continues to increase.

## Introduction

Both chronic hepatitis B (CHB) and non-alcoholic fatty liver disease (NAFLD) are leading causes of liver-related morbidity and mortality, mainly attributable to the development of cirrhosis and hepatocellular carcinoma (HCC). An estimated 296 million individuals are known to have CHB worldwide, with 30% of the global population showing serological evidence of current or past infection (1, 2). The global prevalence of NAFLD is estimated at 38% and continues to increase (3), fuelled by rising rates of obesity and metabolic syndrome.

Modelling studies predict that the prevalence of NAFLD-related cirrhosis and liver mortality will more than double between 2016 and 2030 across multiple regions in the context of an ageing population (4). Additionally, there has been a sharp incline in the proportion of HCC cases attributable to NAFLD, rising from 2.6% between 1995-1999 to 19.5% between the 1990s and 2010s in one European study (5). Despite this, NAFLD has been noticeably absent from global noncommunicable disease strategies, such as the World Health Organisation’s 2023-2030 NCD implementation roadmap (6).

Current antiviral therapies for CHB are non-curative; instead the currently approved agents help prevent disease progression and the consequences of chronic infection by maintaining viral suppression (7, 8). Rates of on-therapy functional cure remain low, with an 8-year cumulative incidence rate ranging from 1.34 to 1.69% in a recent, large multi-ethnic study (9). Paradigms regarding the CHB treatment threshold are changing with compelling evidence of hepatitis B virus (HBV) DNA integration and active inflammation in the hepatitis B e-antigen (HBeAg) positive chronic infection phase (10).

In 2014, the concept of metabolic-associated fatty liver disease (MAFLD) emerged, defined as hepatic steatosis with obesity, diabetes or other evidence of metabolic abnormalities (11). Large-scale studies have demonstrated that MAFLD is associated with an increased risk of all-cause mortality when compared to NAFLD (12, 13). NAFLD/MAFLD is believed to be driven by lipotoxicity, which arises from the increased delivery of free fatty acids to the liver. Subsequent systemic and hepatic inflammation contributes to the development of insulin resistance with assistance from adipokines, bile acids and fibroblast growth factors (14). It has been postulated that HBV infection also contributes to the impairment of insulin signalling via HBx protein, which may interfere with insulin-mediated regulation of gluconeogenic genes (15).

Unlike hepatitis C virus (HCV), HBV does not appear to be overtly steatogenic. A 2011 meta-analysis of 4100 patients with CHB found a similar prevalence of hepatic steatosis in these patients compared to the general population and additionally demonstrated that hepatic steatosis was negatively associated with HBV viral load (16). However, in a cohort of virologically quiescent patients with CHB, the presence of severe hepatic steatosis was found to independently predict fibrosis progression, with a 2-fold increase in rate at 36 months (17). To date, meta-analyses regarding the impact of NAFLD on HBV-related cirrhosis, HCC and mortality have provided conflicting findings (18, 19). Notably, several recent studies have demonstrated the co-existence of MAFLD in patients with CHB to be independently associated with a significantly higher risk of fibrosis (20) and HCC development (21).

Clearly the interactions between these two disease processes are complex and remain poorly defined. Given their risk profiles for the development of complications of chronic liver disease, patients with concurrent CHB and NAFLD may benefit from earlier antiviral treatment to 1) achieve virological suppression and ameliorate fibrosis progression, 2) constrain the tumorigenic mechanisms of early HBV infection, and 3) facilitate their access to trials of novel anti-HBV therapies in the future. NAFLD has been postulated to reduce the efficacy of antiviral CHB treatment, resulting in lower and slower rates of complete virological suppression and biochemical normalisation (22). However, other studies have found that the impact of NAFLD on antiviral response to be negligible (23). The aim of this study was to systematically review the available evidence in this area and provide guidance on the optimal timing for initiating antiviral treatment for patients with concurrent CHB and NAFLD in a clinical setting.

## Methods

### Literature search

We performed a systematic review according to the Preferred Reporting Items for Systematic Reviews and Meta-Analyses (PRISMA) guidelines (24). In order to retrieve all works of potential relevance, a systematic search of the PubMed/Medline and EMBASE databases was performed of all studies through to February 21, 2023. The search used the terms (“Non-alcoholic fatty liver disease” OR “Hepatic steatosis” OR “Steatohepatitis” OR Fatty liver” OR “Metabolic-associated fatty liver disease” OR “NAFLD” OR “MAFLD” OR “NASH”) and (“Chronic hepatitis B” OR “Hepatitis B virus” OR “Hepatitis B infection” OR “CHB” OR “HBV” OR “HBV infection”) AND (“Antiviral treatment” OR “Antiviral response” OR “Antiviral efficacy” OR “Nucleoside analogue” OR “Nucleoside analogue therapy” OR “NA therapy”), which were searched as text words and expanded medical subject headings where possible. The reference lists of relevant articles were also searched for appropriate studies.

### Inclusion criteria

We included randomised or observational studies that met the following inclusion criteria: (1) studies including adult patients with CHB who were on nucleoside analogue therapy with or without concurrent NAFLD, as diagnosed by imaging, controlled attenuation parameter (CAP) or biopsy; (2) studies providing data regarding antiviral efficacy in the form of rates of complete virological suppression, biochemical normalisation and/or HBeAg seroclearance stratified by patients affected by CHB diagnosed with and without NAFLD; (3) studies with a minimum follow-up time of 6 months; (4) studies with a minimum of 50 patients; (5) studies available in English as full papers.

### Exclusion criteria

We excluded studies with (1) populations co-infected with hepatitis C virus (HCV) or human immunodeficiency virus (HIV); (2) populations treated with interferon or other non-nucleoside analogue therapy; (3) populations that had exclusively attained complete virological response; and (4) abstracts only available.

### Data extraction

For each article included, we recorded the authors, year of publication, country of origin, study design, CHB phase and treatment status (eg HBeAg positive and treatment-naïve), NAFLD diagnostic method, frequency of monitoring intervals and duration of follow-up. The baseline characteristics of each study cohort including age, gender, grade of hepatic steatosis, body mass index (BMI), presence of metabolic comorbidities, and levels of total triglycerides, total cholesterol, HBV DNA, alanine transaminase (ALT), aspartate transaminase (AST) and serum quantitative hepatitis B surface antigen (qHBsAg) were extracted. Study outcomes of complete virological response (CVR), biochemical response (BR), and HBeAg loss or seroconversion as defined in each paper (**Table 1**) were recorded at each available monitoring interval.

**Table 1:** Study characteristics.

Paper	Study Type	Location	CHB status	Antiviral	NAFLD Definition	Outcomes of interest
**Jin, 2012** (25)	Prospectiv	Chin	Treatment naïve, HBeAg positiv	ETV	Ultrasoun	CVR (<500 copies/mL) BR (return of ALT to NR) HBeAg loss
**Ceylan, 2016** (26)	Retrospectiv	Turkey	Mixed treatment and HBeAg status	ETV/TDF	Biopsy, Brunt classification	CVR (<20IU/mL)
**Liu, 2016** (27)	Prospectiv	Chin	Treatment naïve, HBeAg positiv	ETV	CT Liver/Spleen Ratio <1	CVR (<500 copies/mL) BR (return of ALT to NR) HBeAg loss
**Zhu, 2016** (28)	Retrospectiv	Chin	Treatment naïve, HBeAg positiv	ETV	Ultrasoun	CVR (<500 copies/mL) BR (return of ALT to NR) HBeAg seroconversion
**Chen, 2017** (29)	Prospectiv	Chin	Treatment naïve, mixed HBeAg status	ETV	CAP >224dB/m	CVR (LLoD unspecifie) BR (return of ALT to NR) HBeAg loss
**Jacobson, 2017** (30)	Prospectiv	Globa	Mixed treatment and HBeAg status	TDF/ADV	Biopsy, NASH Clinical Research Network system	CVR (<69 IU/mL) BR (ALT <43 in M or <34 in F) HBeAg loss
**Kim, 2019** (31)	Retrospectiv	Kore	Treatment naïve, mixed HBeAg status	ETV/TDF	CAP >238dB/m	CVR (<12 IU/mL) HBeAg loss
**Chen, 2020** (32)	Retrospectiv	Taiwan	Mixed treatment status, HBeAg positiv	ETV/TDF ADV/LAM/LdT	Biopsy, Brunt classification	CVR (<20 IU/mL) HBeAg loss
**L, 2020** (23)	Retrospectiv	Americ	Treatment naïve, mixed HBeAg status	ETV/TDF/ADV/LAM/	Imaging (US, MRI, CT) and/or biopsy	CVR (<20-100 IU/mL) BR (ALT <35 in M or <25 in F)
**Tang, 2023** (33)	Retrospectiv	Chin	Treatment naïve, HBeAg positiv	ADV/LdT	Biopsy, NASH Clinical Research System	CVR (<20 IU/mL) BR (return of ALT to NR) HBeAg seroconversion
**Zhang, 2023** (22)	Prospectiv	Chin	Treatment naïve, mixed HBeAg status	ETV/TDF	Biopsy	CVR (<500 copies/mL) BR (return of ALT to NR) HBeAg seroconversion

ADV, Adefovir; ALT, Alanine Aminotransferase; BR, Biochemical Response; CAP, Controlled Attenuation Parameter; CHB, Chronic Hepatitis B; CVR, Complete Viral Response; ETV, Entecavir; F, Female; LAM, Lamuvidine; LdT, Telbivudine; LLoD, Lower Limit of Detection; NAFLD, Non-alcoholic Fatty Liver Disease; M, Male; NR, Normal Range; TDF, Tenofovir Disoproxil.

NB. In Jacobson, 2017; BR in >69 year olds was defined as ALT <35 in M or <32 in F.

### Quality assessment

The Risk of Bias in Non-Randomised Studies of Interventions (ROBINS-I) assessment tool (34) was used to evaluate study quality and is available in the Supplementary Materials. The judgements within each domain of the tool were carried forward to an overall risk of bias judgement, categorised as low, moderate, serious, or critical. Studies judged to be at critical risk of bias were not included in the analysis.

### Statistics

For each comparison, outcomes were compared between two patient groups: patients with CHB who did not have NAFLD (CHB-only group) and patients with concurrent CHB and NAFLD (CHB-NAFLD group). The primary outcomes of interest were the proportion of patients experiencing CVR, BR and HBeAg loss/seroconversion on antiviral therapy. Treating each data point as a single descriptive parameter, the proportions of each outcome were extracted at the following time intervals: 3, 6, 12, 18, 24, 36 and 60 months. In this meta-analysis, results are presented where a minimum contribution of 3 studies to the specific outcome and time interval is met. To stabilise variance, extracted proportions were transformed to logits with calculation of variance thereafter. Pooled logit estimates and corresponding confidence intervals were then back-transformed to proportions by the inverse logit transformation.

Heterogeneity was quantified using the I (2) statistic, where I (2) ≥75.00% indicated substantial heterogeneity, with p < 0.05 defined as the threshold for statistical significance. The τ (2) value was estimated using the restricted maximum likelihood method. Results with substantial heterogeneity are reported in this article but not used to draw conclusions. A random effects model was selected for analyses where heterogeneity was statistically significant (CVR & BR), and a fixed effects model was selected for analyses where heterogeneity was not statistically significant (HBeAg loss/seroconversion) (35). Notably, one study conducted paired liver biopsies on patients before and after antiviral treatment and subdivided them into four categories: sustained non-NAFLD, new-onset NAFLD, sustained NAFLD and remission of NAFLD (33). For the purposes of our meta-analysis, we extracted data from the sustained non-NAFLD and sustained NAFLD patient groups only.

Publication bias was performed on analyses that contained 10 or more studies (36). Assessment was conducted using a combination of statistically significant results in the Egger regression test (37) and subjective asymmetry observed in the funnel plots (**Supplementary Figures 3A–C**). In cases of statistically significant publication bias, fail-safe N calculation was conducted using the General Method (REM) for groups where heterogeneity was statistically significant and the Rosenthal approach for groups where heterogeneity was not statistically significant (38). The value produced was compared to 5n + 10, where n is the number of studies included in the meta-analysis. If the fail-safe N exceeded 5n + 10, then it was determined that the number of missing studies would be unlikely to nullify the result to statistical non-significance.

Meta-analysis results including forest plots and funnel plots were performed using Review Manager 5.4 (39). Publication bias tests, as detailed above, were performed using the regtest() function and fsn() function in the metafor package in R (40).

## Results

### Characteristics of included studies

The search identified 470 titles and abstracts that were reviewed, with 32 citations being selected for full-text review. Of these, 21 studies were excluded after rigorous review, as demonstrated in the study selection flowchart (**Supplementary Figure 1**). In total, seven studies included patients on interferon or other non-nucleoside analogue therapy for CHB. The full text in English for 12 studies could not be sourced despite cross checking databases; one study did not provide data in the form required regarding our outcomes of interest; and one study entailed a CHB population that had exclusively attained CVR. Therefore, 11 studies comprising 2580 patients were included in the analysis (22, 23, 25–33). 1630 patients had CHB only, while 950 patients had concurrent CHB and NAFLD.

Five studies consisted of CHB populations that were exclusively HBeAg-positive, while six studies comprised both HBeAg-positive and HBeAg-negative patients. Eight of 11 studies specified that the enrolled CHB populations were previously treatment-naïve. The presence of NAFLD was defined by biopsy in five studies, CAP parameter in two studies, ultrasound in two studies, computed tomography (CT) findings in one study and by a choice of either imaging (ultrasound, CT or magnetic resonance imaging) or histology in one study. Definitions of CVR varied between studies, from an HBV DNA <12 IU/mL to an HBV DNA <500 copies/mL. Certain studies specified an ALT range to qualify as BR, while other studies defined BR as a return of ALT to “normal range”. Six studies reported on HBeAg loss while three studies reported on HBeAg seroconversion; these outcomes were combined in our meta-analysis. Follow-up varied between 12 to 60 months. Eight studies were based in Asia, one study was based in America, one study was based in the Middle East, and there was just one global study. All study characteristics are summarised in **Table 1**.

### Complete virological response

**Figure 1:**
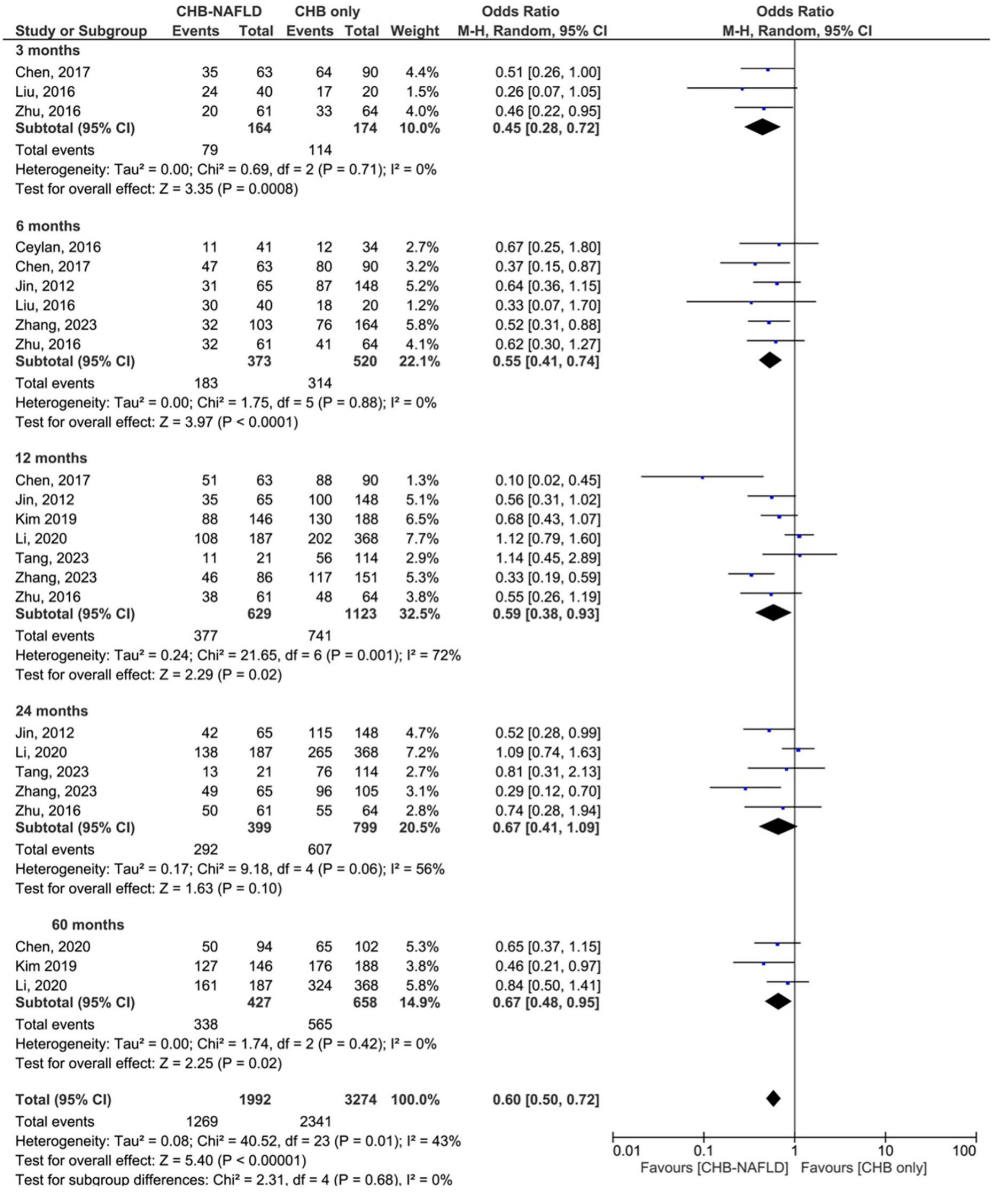
Comparison of complete virological response (CVR) between CHB patients with and without NAFLD.

Patients with CHB-NAFLD demonstrated significantly lower rates of complete virological response than patients in the CHB-only group at almost all timepoints. The presence of NAFLD reduced rates of CVR by odds ratios of 0.45 (0.28-0.72, p=0.0008, I^2^=0%) at 3 months, 0.55 (0.41-0.74, p<0.0001, I^2^=0%) at 6 months, and 0.59 (0.38-0.93, p=0.001, I^2^=72%) at 12 months. At 24 months, CHB-NAFLD patients tended towards lower rates of CVR compared to CHB-only patients with an odds ratio of 0.67 (0.41-1.09, p=0.10, I^2^=56%), but this result did not reach statistical significance. Finally at 60 months, the presence of NAFLD reduced rates of CVR by an OR of 0.67 (0.48-0.95, p=0.02, I^2^=0%), thus demonstrating a diminishing effect size over time. The number of contributing studies was highest for the 12-month timepoint (7 studies), as demonstrated in **Figure 1**. Overall significance was calculated at p<0.00001 and overall heterogeneity was calculated at I^2^=43%.

### Biochemical response

**Figure 2:**
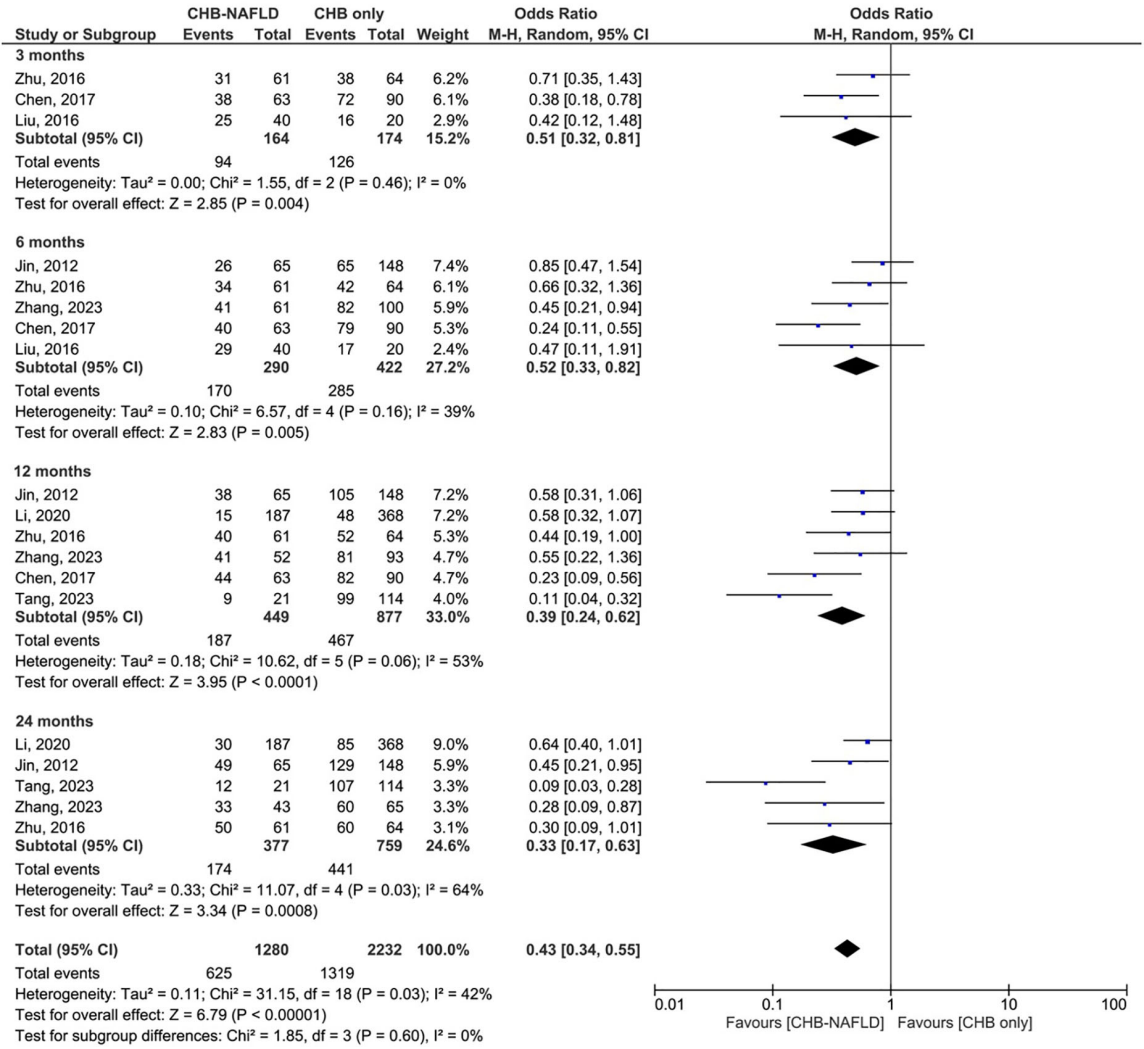
Comparison of biochemical response (BR) between CHB patients with and without NAFLD.

Patients with CHB-NAFLD demonstrated significantly lower rates of biochemical response compared to the CHB-only group at all timepoints. The presence of NAFLD reduced rates of BR by odds ratios of 0.51 (0.32-0.81, p=0.004, I^2 =^ 0%) at 3 months and 0.52 (0.33-0.82, p=0.005, I^2 =^ 39%) at 6 months. At later timepoints, CHB-NAFLD patients demonstrated progressively lower rates of BR in comparison to CHB-only patients, with odds ratios of 0.39 (0.24-0.62, p<0.0001, I^2^ = 53%) at 12 months and 0.33 (0.17-0.63, p=0.0008, I^2^ = 64%) at 24 months respectively, thus demonstrating a strengthening effect size over time. The number of contributing studies was highest for the 12-month timepoint (6 studies), as demonstrated in **Figure 2**. Overall significance was calculated at p<0.00001 and overall heterogeneity was calculated at I^2^ = 42%.

### HBeAg loss/seroconversion

While patients with CHB-NAFLD tended towards lower rates of HBeAg loss/seroconversion compared to the CHB-only group, there were no significant differences between groups at any timepoint. A comparison of HBeAg loss/seroconversion rates between the CHB-NAFLD group against the CHB-only group yielded ORs of 0.70 (0.40-1.24, p=0.23, I^2^ = 0%) at 6 months, 0.87 (0.56-1.35, p=0.52, I^2^ = 0%) at 12 months, and 0.78 (0.51-1.20, p=0.26, I^2^ = 0%) at 24 months respectively. The number of contributing studies was 4 studies for the 6- and 24-month timepoints and 5 studies for the 12-month timepoint, as demonstrated in **Supplementary Figure 2**. Overall significance was calculated at p=0.10 and overall heterogeneity was calculated at I^2^ = 0%.

## Discussion

Our meta-analysis illustrates that patients with concurrent CHB and NAFLD demonstrate significantly lower CVR rates in the short-term, with the difference between CHB-only and CHB-NAFLD patient groups tapering off over time. Accordingly, Zhang et al (22) demonstrated that there was a significant difference between the time to CVR between CHB-only and CHB-NAFLD groups, at 6 months (95% CI 3-10 months) vs 8 months, (95% CI 4-14.5 months), p=0.039. While early viral response rates at 3 and 6 months may predict the likelihood of sustained remission, comparing CVR rates at 12 months and beyond between CHB-only and CHB-NAFLD patient groups may be more clinically relevant in allowing the full impact of antiviral therapy to be realised. Our results at all timepoints suggest that current antiviral therapies may take longer to suppress viral replication in patients with concurrent CHB and NAFLD. This is a clinically significant finding given that on-therapy low-level viraemia has previously been associated with higher risks of HCC development (41). A more proactive approach towards initiating antiviral treatment in CHB-NAFLD patients may therefore contribute to reducing the sequelae of chronic liver disease.

Furthermore, our meta-analysis confirms that patients with CHB-NAFLD demonstrate a different on-treatment biochemical profile compared to patients with CHB only. The presence of NAFLD in virologically suppressed patients with CHB on long-term therapy has previously been shown to correlate with incomplete BR (30, 42), which may represent a combination of the concurrent disease process and the effects of persistent viraemia in this cohort. In fact, Jacobson et al (30) found that the presence of hepatic steatosis was significantly associated with increased ALT levels at 5 years, with a multivariate OR of 2.236 (1.031-4.852, p=0.042). While incomplete biochemical response in these CHB-NAFLD patients can, at the very least, be partially attributed to the NAFLD disease process itself, it is well established that those patients with persistent ALT elevations, regardless of aetiology, are at risk of steatosis progression (30) and HCC development (42, 43). This is in keeping with the growing body of evidence that HBV disease stages previously not considered for antiviral therapy are also at risk of disease progression with fibrosis development and hepatocarcinogenesis.

The impact of hepatic steatosis on antiviral efficacy is likely distinct from that of associated host metabolic factors, such as hyperlipidaemia and insulin resistance. Almost all included studies recruited patients with CHB, with and without NAFLD, who were well matched in terms of age, gender, baseline ALT, HBV DNA and even total cholesterol levels (**Table 2**). However, there were unsurprisingly significant differences in BMI and serum triglyceride levels between groups. Despite this, 3 of 4 studies that sought to determine independent risk factors for CVR produced significant multivariate HRs when considering hepatic steatosis (22, 25, 31). For example, Zhang et al. demonstrated an independent association between hepatic steatosis and CVR at 24 months (22). This relationship strengthened with severity of steatosis, culminating in a MV HR of 0.085 (0.019-0.392, p=0.002) for patients with Grade 3 steatosis vs no steatosis. Furthermore, obesity has not been shown to impact CHB antiviral efficacy in patients with CHB (44), which further supports an independent relationship between hepatic steatosis and CVR, as explored in our meta-analysis. Nevertheless, the co-existence of metabolic syndrome and CHB has been associated with higher rates of ALT elevation (45), advanced liver fibrosis and delayed HBeAg seroclearance (46). Adipokines, such as leptin and fibroblast growth factor 21, are associated with metabolic dysregulation and correlate with advanced fibrosis/cirrhosis in CHB patients on treatment (47, 48). Furthermore, HCC risk in the CHB-MAFLD patient cohort is directly influenced by host metabolic factors, such as obesity and diabetes (49). In essence, multimorbidity serves as a poor prognosticator for the sequelae of CHB-related chronic liver disease and should be factored into decisions regarding the timing of antiviral treatment.

**Table 2:** Patient characteristics.

Paper	# Patients		Age		# Male		BMI		TG (mmol/L)		TC (mmol/L)		ALT (U/L)		AST (U/L)		HBV DNA (log 10 copies/mL)		qHBsAg (log 10 IU/mL)	
	*#1*	*#2*	*#1*	*#2*	*#1*	*#2*	*#1*	*#2*	*#1*	*#2*	*#1*	*#2*	*#1*	*#2*	*#1*	*#2*	*#1*	*#2*	*#1*	*#2*
**Jin, 2012** (25)	148	65	39.6	39.6	85	32	**24.3**	**26.4**	**1.1**	**1.5**	4.4	4.5	159	172	57	60	6.7	6.7	.	.
**Ceylan, 2016** (26)	41	34	**33**	**46**	.	.	**23**	**29**	2.2	2.4	**4.3**	**4.8**	106	81	.	.	5.4	4.8	.	.
**Liu, 2016** (27)	20	40	37.4	37.7	12	23	**22.1**	**28.1**	**1.4**	**2.7**	**4.5**	**6.4**	230	228	168	183	7.4	7.2	2.7	2.7
**Zhu, 2016** (28)	64	61	39.6	39.3	41	42	**22.5**	**26.3**	**1.3**	**1.8**	3.7	3.8	188	180	.	.	6.2	6.2	.	.
**Chen, 2017** (29)	90	63	41.5	43.1	70	53	.	.	.	.	.	.	102	130	65	72	5.2	5.4	.	.
**Jacobson, 2017** (30)	339	128	.	.	.	.	.	.	.	.	.	.	.	.	.	.	.	.	.	.
**Kim, 2019** (31)	188	146	51	51	119	91	**22.5**	**24.7**	.	.	**4.4**	**4.6**	56	56	.	.	5.7	6.1	.	.
**Chen, 2020** (32)	94	102	**36.2**	**42.7**	61	81	**22.7**	**25.2**	.	.	.	.	121	102	65	62	7.9	7.5	**4**	**3.7**
**Li, 2020** (23)	368	187	45.5	47.7	**211**	**126**	**23.8**	**25.4**	**2.2**	**4.9**	4.6	4.7	50	60	35	37	4.6	4.5	.	.
**Tang, 2023** (33)	114	21	29	29	91	21	20.7	24.4	.	.	.	.	143	142	84	69	8.1	8.3	4.3	4.5
**Zhang, 2023** (22)	164	103	37	36	111	75	**23.4**	**25.7**	.	.	.	.	62	55	41	34	6.6	6.9	3.7	4
**Pooled results**	1630	950	41.3	43.5	64%	69%	**23.4**	**25.7**	**1.8**	**3.4**	4.4	4.7	93	98	53	58	6.0	6.1	3.9	3.7

ALT, Alanine Aminotransferase; AST, Aspartate Aminotransferase; BMI, Body Mass Index; qHbsAg, Quantitative HBsAg level; TC, Total cholesterol; TG, Total triglycerides; “#”, Number of; “.”, Data not provided.

NB 1. Group #1 refers to CHB-only patients; Group #2 refers to CHB-NAFLD patients.

NB 2. Bolded values indicate a significant difference between CHB-only and CHB-NAFLD groups (p<0.05).

NB 3. Jacobson, 2017 did not provide baseline characteristics distinguished by CHB-only and CHB-NAFLD groups.

NB 4. Tang, 2023 did indicate significance regarding the difference in baseline characteristics between CHB-only and CHB-NAFLD groups.

High controlled attenuation parameter (CAP) values early in the CHB disease course may serve as another indication for proactive treatment to avert progression to fibrosis and other complications of chronic liver disease. Compared to ultrasound and hepatic steatosis index, CAP on transient elastography has emerged as the method with superior diagnostic accuracy, particularly in detecting mild steatosis (50). While CAP measurements of steatosis have previously been shown to correlate well with severity of fibrosis in patients with CHB (51), emerging data suggests that CAP values should be carefully interpreted in the context of severe fibrosis and transition to cirrhosis. The hepatic fat loss in advanced fibrosis and cirrhosis is attributed to portal hypertension, changes in vasculature and chronic inflammatory state. This is why lower CAP values in the context of high liver stiffness measurements (LSM) have been linked to higher rates of HCC, while the prognostic implications are the reverse in patients without advanced chronic liver disease (52). A CAP value > 280 dB/m, denoting the presence of severe steatosis, has previously been shown to predict fibrosis progression in a CHB-NAFLD population (17) suggesting it could be considered as a threshold value to trigger initiation of antiviral therapy in these patients.

Two recent meta-analyses have published conflicting results regarding the impact of concomitant hepatic steatosis on rates of HBV-related cirrhosis and HCC (18, 19). However, the severity of steatosis was not accounted for in the analyses, and as previously suggested, this may be key in predicting those patients most likely to develop advanced fibrosis. From this perspective, the timeline to develop cirrhosis and HCC is well established. Given that death rates from HBV-related HCC are expected to double by 2040 (53), any factors that may exacerbate HCC risk should be carefully analysed. Interestingly, both meta-analyses, alongside other studies, support an association between the presence of NAFLD and higher rates of functional cure (17–19, 54, 55). Hypotheses proposed for this finding include the alteration of HBsAg cytoplasmic distribution by abnormal lipid metabolism in patients with CHB-NAFLD, and overexpression of Fas receptor contributing to hepatocyte apoptosis (56). Nonetheless, HBsAg seroclearance is a rare event that usually occurs late in the course of infection and absolute rates of functional cure remain low in patients with treated CHB-NAFLD. For example, Hsu et al’s study demonstrated a cumulative rate of under 4% over 10 years despite being about twice as likely to achieve this outcome compared to patients without fatty liver, (adjusted HR 1.97, 1.09-3.55, p=0.02) (54). Ultimately, given that the body of evidence supporting the association between NAFLD and functional cure encompasses both treated and untreated populations, this would not serve as an argument against initiating antiviral therapy in this cohort of patients.

The heterogeneity of some findings in the meta-analysis owes partially to the difference in NAFLD diagnostic methodologies between studies. Additionally, all studies bar one categorised patients as CHB-only or CHB-NAFLD based on one diagnostic measurement at the beginning of the study; thus closer monitoring of NAFLD may be necessary. Furthermore, all included studies recruited patients with the traditional criteria for NAFLD rather than the newly conceptualised MAFLD, which will likely come to the forefront in future study designs. Other weaknesses included the uneven spread of data points, with sparse evidence to support outcomes at 3 to 5 years given that most studies had a maximum of 24 months of follow-up. For the same reason, we did not attempt to quantify the impact of NAFLD on other longer-term outcomes such as functional cure, cirrhosis, decompensation, and HCC in this cohort of treated CHB patients. Moreover, most studies had exclusively HBeAg positive populations and studies with mixed populations did not publish their findings categorised by HBeAg status. Finally, given that a significant proportion of studies are from Asia with distinct mediating epidemiological HBV patterns, genotype and genetic influences should be considered, especially when interpreting HBeAg loss/seroconversion data.

Treatment thresholds in CHB are changing across all the major liver disease organisations and may lead to broadening treatment candidacy for these patients. With emerging cost-effective analyses supporting this aim, patients will likely be offered antiviral therapy to avert the complications of CHB, specifically the development of HCC (57, 58). The timing of initiating antiviral treatment in CHB is guided by a complex interplay between viral and immune dynamics, and the outcomes explored in our meta-analysis represent just one facet of the decision to treat process. However, the rationale for a more proactive approach and earlier intervention in CHB treatment is gaining momentum in the field, and the risk of liver disease progression is likely higher in multimorbid patients who demonstrate a slower path to CVR or in those subjects with persistent ALT elevations. Patients with CHB-NAFLD who do not achieve a satisfactory response to standard antivirals should be considered for novel therapies, given the heightened risk of progression to fibrosis, cirrhosis, and HCC. Future studies should examine the impact of NAFLD, in isolation and with other metabolic risk factors, on antiviral efficacy in patients across all CHB disease phases over longer follow-up times.

## Data availability statement

The original contributions presented in the study are included in the article/**Supplementary Material**. Further inquiries can be directed to the corresponding author.

## Author contributions

GZ: Conceptualization, Data curation, Methodology, Writing – original draft. BH: Formal analysis, Writing – review & editing. SA: Supervision, Writing – review & editing. UG: Supervision, Writing – review & editing. PK: Conceptualization, Supervision, Writing – review & editing.
