# Supplementary material for: The co-existence of NAFLD and CHB is associated with suboptimal viral and biochemical response to CHB antiviral therapy: a systematic review and meta-analysis
Source: Front Gastroenterol (Lausanne). 2024 Jan 24;3:1333988. doi: 10.3389/fgstr.2024.1333988 (PMC12952471; doi:10.3389/fgstr.2024.1333988)
Supplement: Supplementary file 1 [file DataSheet_1.docx]

| Paper | D1 | D2 | D3 | D4 | D5 | D6 | D7 | Overall |
| --- | --- | --- | --- | --- | --- | --- | --- | --- |
| Jin, 2012^38^ | M | L | L | L | M | L | L | L |
| Ceylan, 2016^39^ | M | L | L | L | M | L | M | M |
| Liu, 2016^40^ | M | L | L | L | L | L | L | L |
| Zhu, 2016^41^ | M | L | L | L | L | L | L | L |
| Chen, 2017^42^ | L | L | L | L | L | M | L | L |
| Jacobson, 2017^43^ | M | M | L | L | L | L | L | L |
| Kim, 2019^44^ | M | L | L | L | L | L | L | L |
| Chen, 2020^45^ | M | L | L | L | L | L | L | L |
| Li,  2020^28^ | M | L | L | L | M | L | L | L |
| Tang, 2023^32^ | M | M | L | L | L | L | L | L |
| Zhang, 2023^27^ | M | M | L | L | L | L | L | L |

**Supplementary Table 1. Risk of Bias Assessment in Studies**

L = Low, M = Moderate, S = Serious, C = Critical.

Bias Domains included in the Robins-I tool

D1: Bias due to confounding
D2: Bias in selection of participants into the study
D3: Bias in classification of interventions
D4: Bias due to deviations from intended interventions
D5: Bias due to missing data
D6: Bias in measurement of the outcome
D7: Bias in selection of the reported result

**
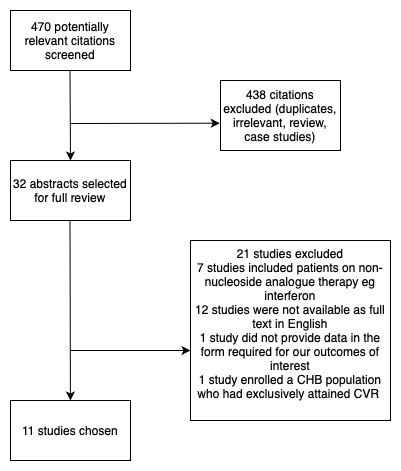
**

**Supplementary Figure 1. Study Selection Flowchart**

*
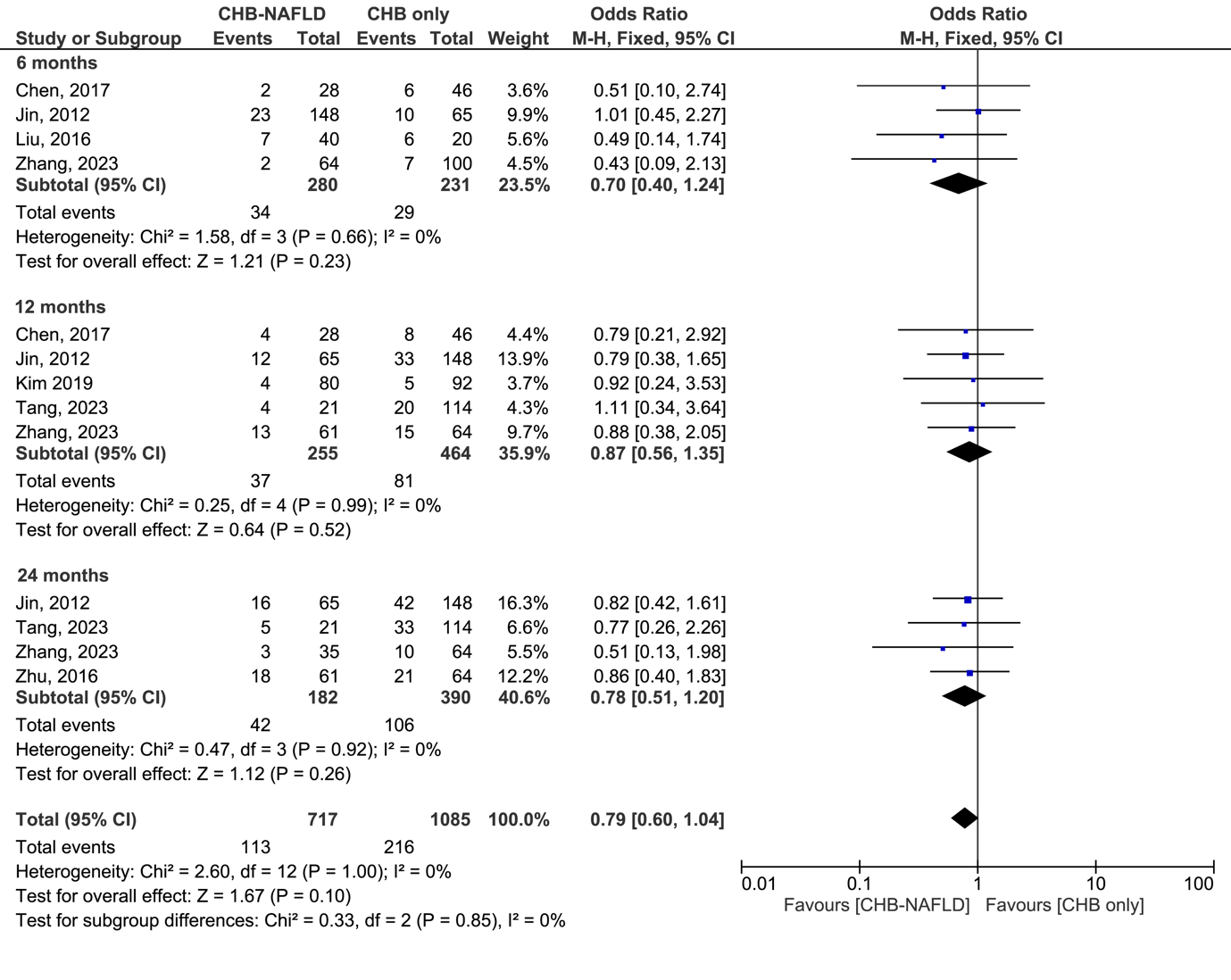
*

**Supplementary Figure 2. Comparison of HBeAg loss/seroconversion between CHB patients with and without NAFLD**

*
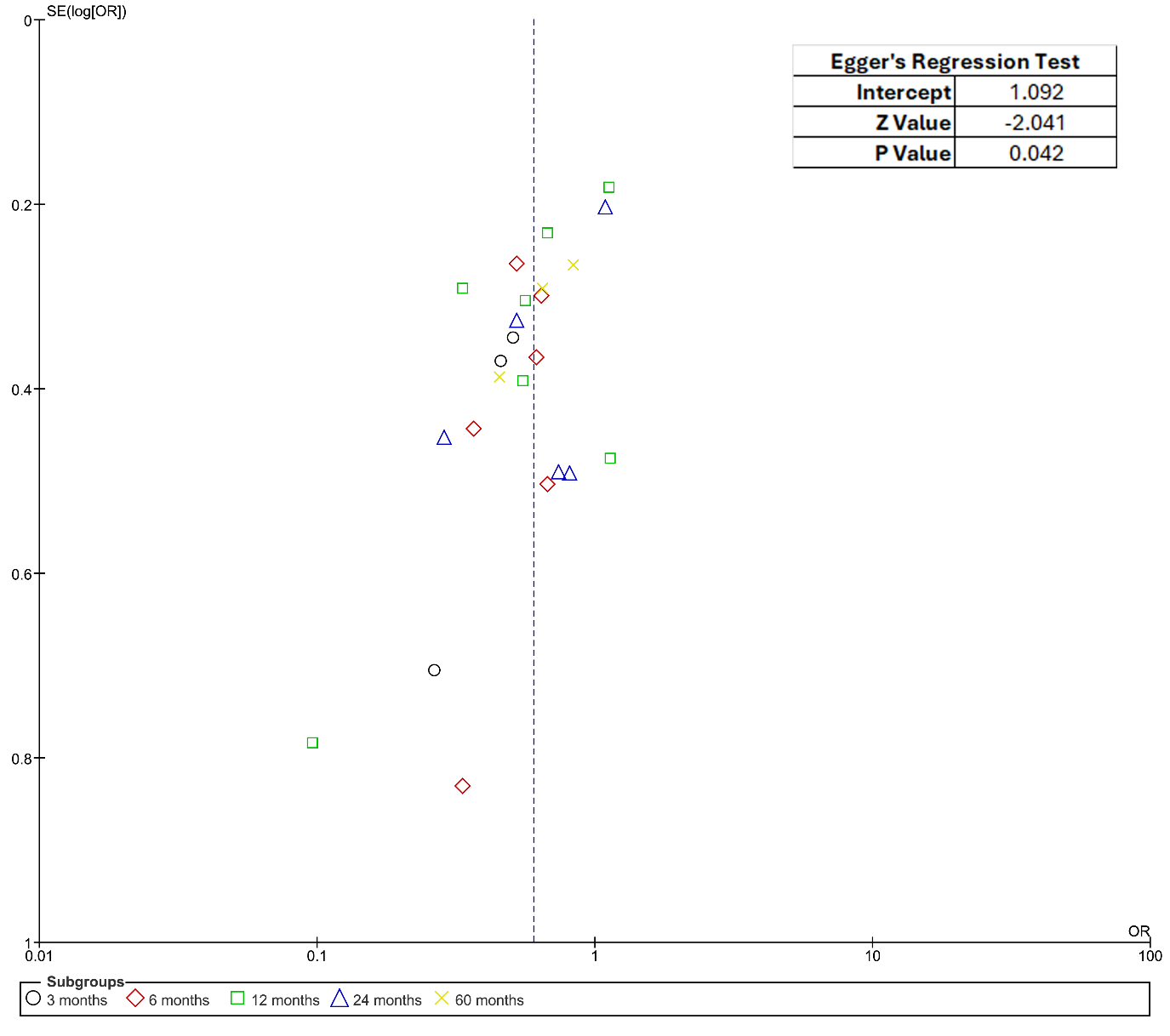
*

**Supplementary Figure 3a. Funnel plot for the comparison of complete virological response (CVR) between CHB patients with and without NAFLD**

*NB: Fail-safe N calculation using the REM approach = 273*

*
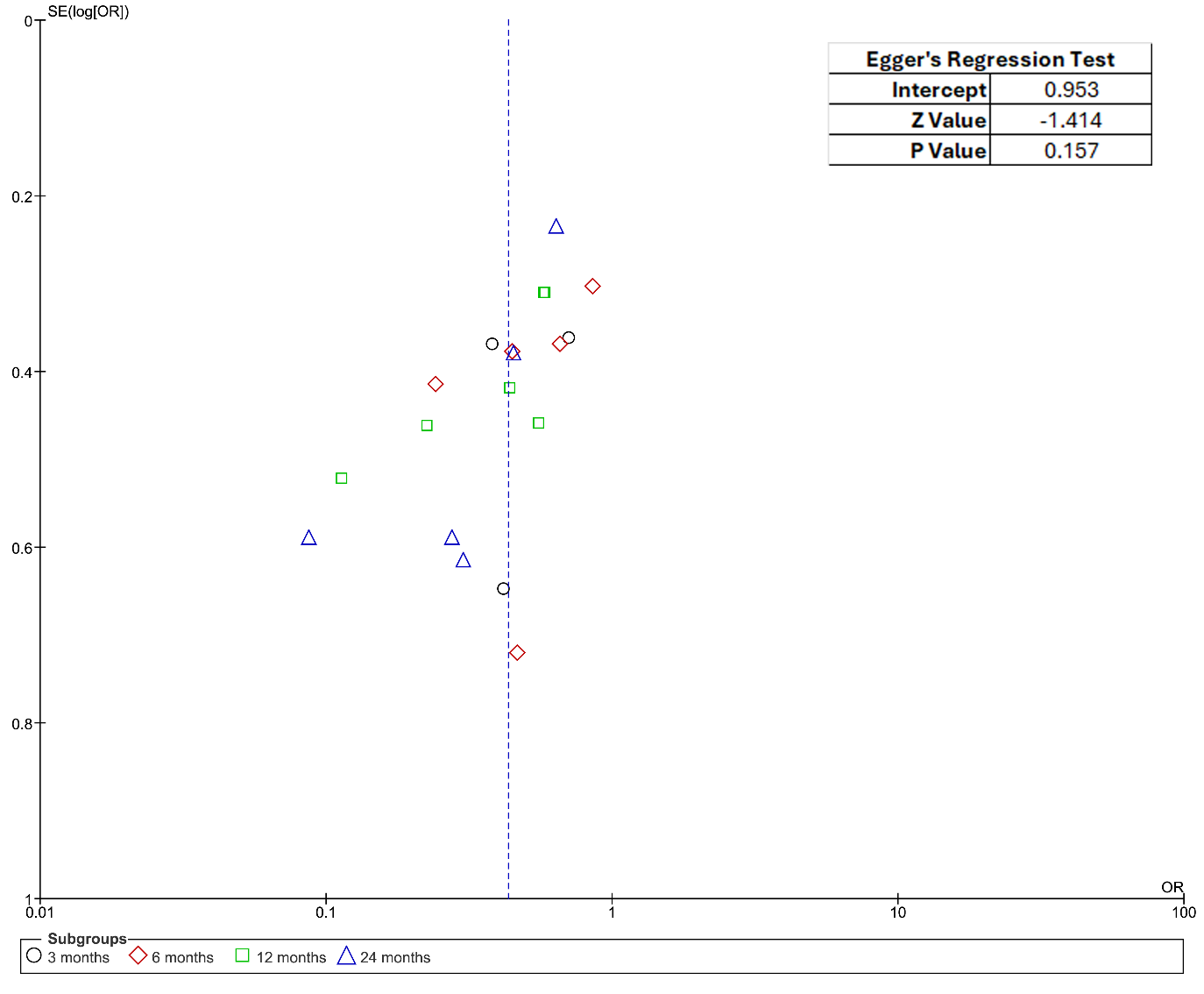
*

**Supplementary Figure 3b. Funnel plot for the comparison of biochemical response (BR) between CHB patients with and without NAFLD**


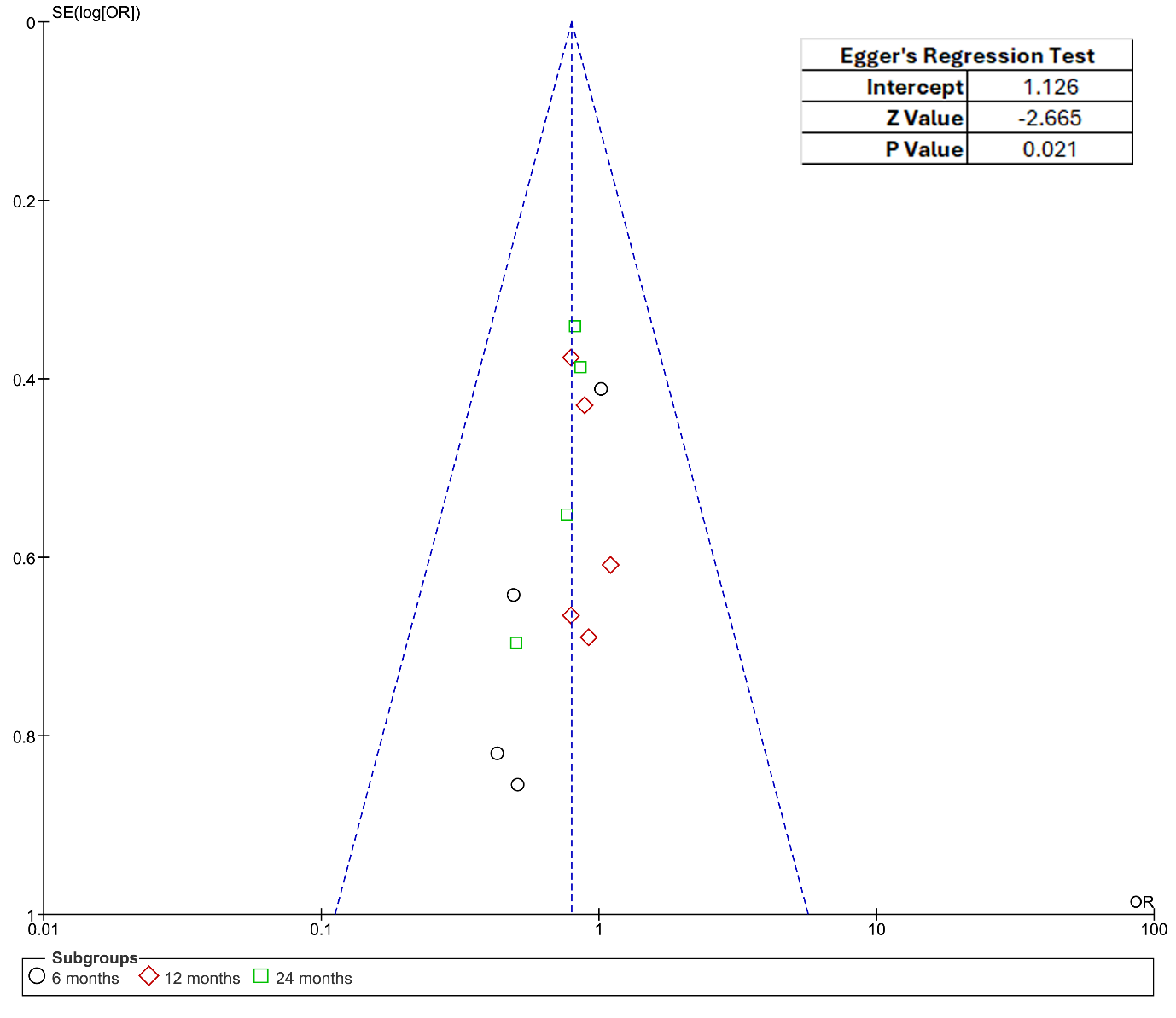


**Supplementary Figure 3c. Funnel plot for the comparison of HBeAg loss/seroconversion between CHB patients with and without NAFLD**

*NB: Fail-safe N calculation using the Rosenthal approach = 130*
